# Mode III Tear Resistance of *Bombyx mori* Silk Cocoons

**DOI:** 10.1021/acsmaterialsau.4c00001

**Published:** 2024-03-28

**Authors:** Ateeq Ur Rehman, Vasileios Koutsos, Parvez Alam

**Affiliations:** School of Engineering, Institute for Materials and Processes, The University of Edinburgh, Robert Stevenson Road, Edinburgh EH9 3FB, U.K.

**Keywords:** *Bombyx mori*, silk cocoon, tear resistance, tear strength, mode III failure, textiles, cocoon damage

## Abstract

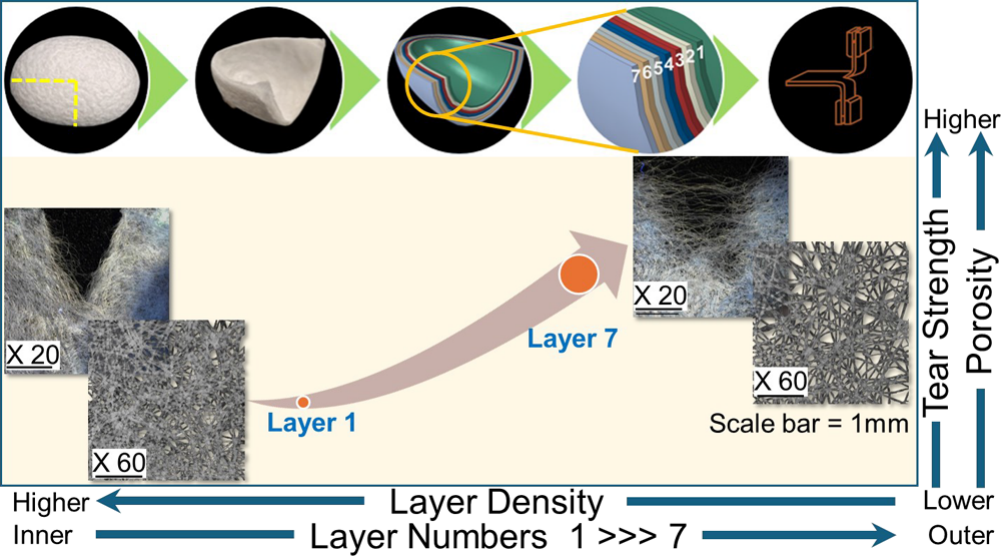

This paper concerns the tear properties and behavior of *Bombyx mori* (*B. Mori*) silk cocoons. The tear resistance of cocoon layers is found to increase progressively from the innermost layer to the outermost layer. Importantly, the increase in tear strength correlates with increased porosity, which itself affects fiber mobility. We propose a microstructural mechanism for tear failure, which begins with fiber stretching and sliding, leading to fiber piling, and eventuating in fiber fracture. The direction of fracture is then deemed to be a function of the orientation of piled fibers, which is influenced by the presence of junctions where fibers cross at different angles and which may then act as nucleating sites for fiber piling. The interfaces between cocoon wall layers in *B. mori* cocoon walls account for 38% of the total wall tear strength. When comparing the tear energies and densities of *B. mori* cocoon walls against other materials, we find that the *B. mori* cocoon walls exhibit a balanced trade-off between tear resistance and lightweightness.

## Introduction

*Bombyx mori* (*B. mori*) is one of the most useful domesticated species of mulberry silkworms,^1,2^ spinning a silk cocoon to protect itself as it develops morphologically from its pupal stage. Silk fibers have been used in textiles for more than 4000 years,^3^ with the earliest example of a woven silk fabric originating in 3630 BC.^4^ In a typical silk production process, the cocoon is degummed to separate sericin proteins (the glue) from fibroin proteins (the structural thread). Silk fibers are strong yet soft to the touch, flexible, durable,^5^ and absorbent to a wide variety of dyes.^1^

During cocoon construction, *B. mori* extrudes silk fibers through the spinneret in its mouth.^6^ Similar to silk egg cases,^7−13^ the *B. mori* cocoon has a protective function.^14−19^ It is a sophisticated multilayer nonwoven composite comprised of continuous silk fibers connected by a sericin gum-like coating that acts as a microstructural level glue for the fibers.^20^*B. mori* cocoon size ranges from 30 to 35 mm.^2^ A typical cocoon has been reported as having a meridional diameter of 31.57 ± 0.19 mm and an equatorial diameter of 19.01 ± 0.17 mm.^21^ Its wall is typically 0.30–0.59 mm thick,^14,19,20,22,24^ it has a density between 377 and 499 kg/m^3^,^19,20^ and contains 70–80% silk fibroin with the remainder being sericin protein.^3,25^ Silk fiber has a triangular or irregular cross-section with an approximated diameter between 16 and 26 μm. A single fiber consists of two fibroins, each with a diameter of 7–14 μm, which are conjoined by a thin sericin layer 2–4 μm in thickness.^14,24,26^

The cocoon wall of *B. mori* can be divided into 5 to 15 distinct layers, each varying in their sericin to fibroin compositional ratio and each exhibiting different microstructures. The innermost layers are comprised of fibers with mean diameters of ca. 16 μm and the number of fibers/mm^2^ is ca. 21. Contrarily, the outermost layers have higher mean fiber diameters of ca. 26 μm but there are only 8 fibers/mm^2^ in these layers.^14^ Comparatively, the inner layers of *B. mori* cocoons comprise lower fractions of sericin, but stronger levels of interfiber bonding.^22,27,28^ The density of *B. mori* layers has been reported as decreasing from the innermost to the outermost layer.^29^ It has additionally been noted that porosities between the inner and outer layers differ significantly and are reported in the ranges of 0.42–0.70 and 0.58–0.84, respectively.^30^ The density of silk fiber ranges between 1350 and 1365 kg/m^3^.^29^

Tensile strength, tensile modulus, and the toughness of the cocoon wall are of the order: 16.6–54 MPa, 300–586 MPa, and 1.1 MJm^–3^, respectively.^22,24,31,32^ The wall will strain 18 ± 2% at maximum tensile strength and the breaking strain is typically in the order of 13–35%.^22,32^ Additionally, the elliptical design of silk cocoons has been understood as being of significant benefit to impact damage tolerance, as an ellipsoid can elastically deform on impact in such a way that energy is stored in the ellipsoid and released at a critical level proportional to the impact force.^33^

The hierarchical structure of *B. mori* silk cocoon impacts the mechanical properties of the subdivided layers.^19,34,35^ Young’s modulus, tensile strength, storage modulus, and loss modulus are all reported as being higher in the inner pelade layer (near the pupa) than they are in the thickness-averaged values of the complete cocoon.^36^ The strength and modulus of individual layers rise as fiber areal density increases, and as porosity and fiber diameter decrease from the outermost to the innermost layers.^24^ The specific modulus and specific strength of the innermost layer have been reported as being the highest at 24 GPa mm^–1^ and 938 MPa mm^–1^, respectively, while they are significantly lower in the outermost layer, at 1.6 GPa mm^–1^ and 159.4 MPa mm^–1^, respectively. Contrarily, strain at peak stress is the lowest in the innermost layer (7.9%) and highest at the outermost layer (21.3%). Each cocoon layer contributes to its mechanical properties and behavior, and the layers are held together mostly by sericin as well as by a few cross-linking fibers. As such, interlayer bonding within a cocoon wall is significantly weaker than intralayer bonding.^22^

The cocoon wall is also permeable to moisture, and moisture flux in the outer layers is higher than in the inner layers, as the inner layers are generally lower in porosity and are of higher tortuosity.^30^ In addition, the cocoon wall is extremely permeable to gases under normal conditions, yet it has the capacity for CO_2_ gating and thermoregulation under more extreme environmental conditions,^37,38^ and is thus able to maintain its internal temperature and CO_2_ levels. It is therefore clear that one of the benefits of the distinctive design of the cocoon wall is to offer protection to the resident pupa, whether that be mechanical, gaseous, or thermal.^14^

While the structure–property relationships of a variety of silk fibers have been researched extensively,^26,39−44^ knowledge of silk cocoons is more sparsely documented. As mentioned, cocoons are structures that provide protection to pupae, and herein we hypothesize that the geometrically complex, hierarchical, and layered structure of a *B. mori* cocoon should provide a certain level of tolerance to tearing. This is because mice and other small mammals (e.g., *Peromyscus leucopus noveboracensis*, *P. maniculatus bairdii*, *Mus musculus*, *Microtus ochrogaster*, and *Blarina brevicauda*) are able to prey on silkworm pupae by tearing through the cocoon wall.^45^ Multiple reports document the tear resistance of silk sheets and silk derived/inspired composites,^46−50^ yet as far as we are aware, there has been no work conducted to date on the tear strength of cocoon walls and their layers. Knowledge of this property will enable a more detailed understanding of how cocoons mechanically protect their pupae. As such, this paper aims to fill this knowledge gap and we aim to detail the tear strength of *B. mori* silk cocoons, in relation to their structures.

## Materials and Methods

### Tear Testing of Full Cocoon Walls

**Figure 1:**
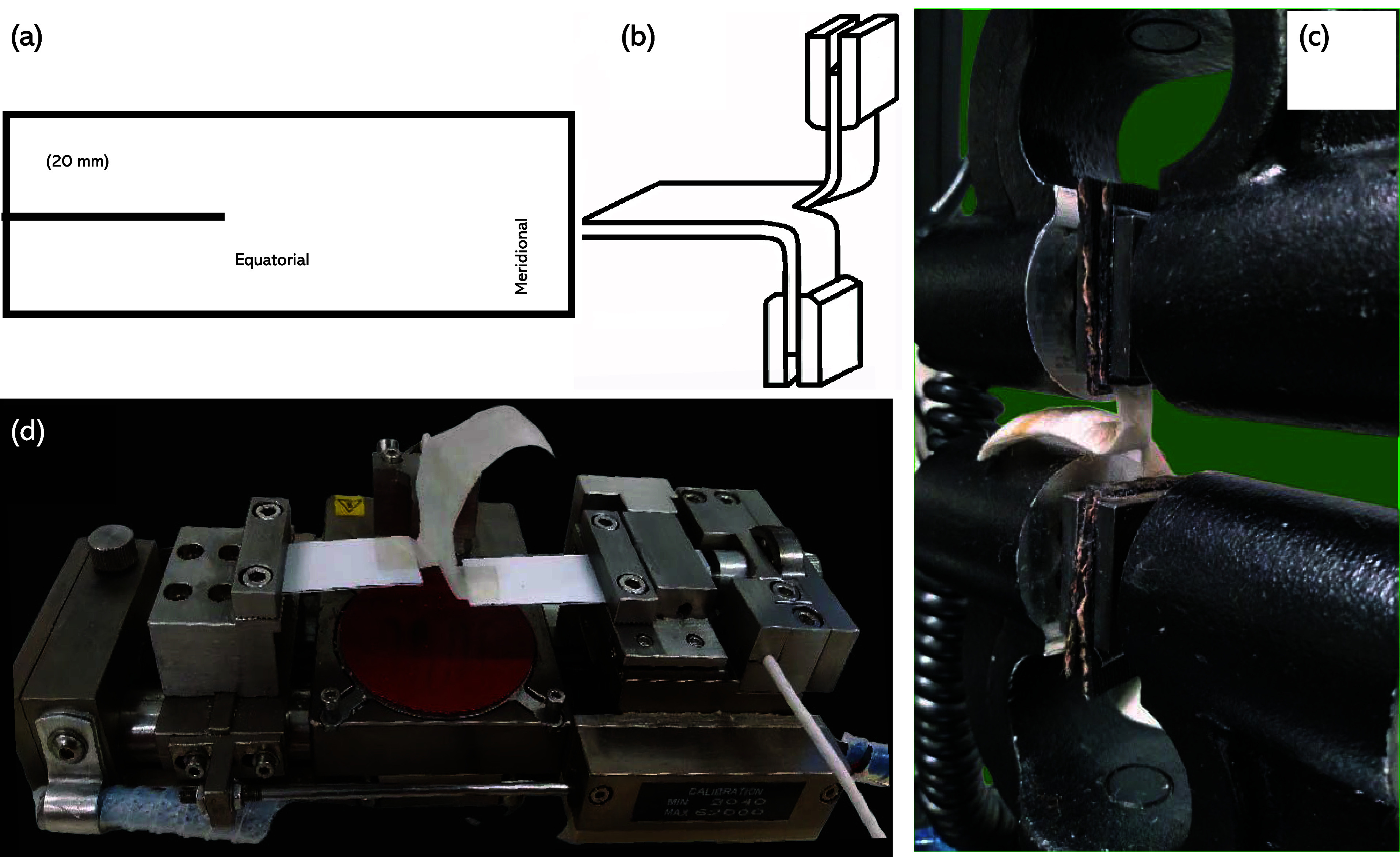
(a) Trouser test full cocoon specimen, (b) trouser tearing, (c) Instron 3369 experimental setup, and (d) Deben microtensile tester experimental setup.

A modified ASTM D624-00^51^ trouser tear test method was used to enable testing of the smaller-than-standard-sized samples (due to restrictions imposed by the sizes of the *B. mori* cocoons). *B. mori* cocoons were purchased from the Kabondo Silk Factory and Marketplace, Kisumu, Kenya. Cocoon macro measurements were made using a Vernier caliper, and thickness measurements were made using a digital screw gauge. Thirty rectangular trouser tear samples were prepared (*n* = 30) from 30 individual cocoons by cutting a 20 mm slit in the equatorial direction of the cocoon wall (Figure 1a). The open cocoon walls were tear tested using an Instron 3369 (High Wycombe, UK) with a 1 KN load cell. The initial gauge length was 10 mm, and testing was conducted at a displacement rate of 10 mm/min. Each of the split halves of the individual sample (i.e., the trouser legs) was held in the Instron grip and was aligned with the center line of the sample. The uncut end of the specimen was kept free, and this is the part that would experience a tear at a right angle to the line of the force application. Figure 1b provides a schematic of the trouser tear test, and Figure 1c shows the experimental setup. All tests were conducted at 65% relative humidity and 21 °C.

### Tear Testing of Individual Cocoon Layers

Cocoon walls were separated into seven individual layers. Although individual cocoon walls can in principle be separated into up to 15 layers, these layers are extremely thin and difficult to test mechanically. As such, we employed a similar approach to Chen et al.^22^ who subdivided the cocoon into seven layers to enable layer-by-layer tensile testing. The seven individual layers were essentially therefore comprised of adjacent layers within the cocoon wall. We used thickness measurements and one-way ANOVA analysis between the thickness measurements to help ascertain that the layers were the same. Our assumption is that the subdivided layers should not show any difference in thickness. Using an α = 0.05 we note that the *p*-value of 0.34, which is above α. As such, we concluded that there was no significant difference between the mean thickness of selected sets of layers, and we used these for further testing. These cocoon layers (hereinafter: individual layers) were then cut into 20 mm wide strips. At the end of each sample, a 10 mm long slit was made in the equatorial direction at the center fold line of the sample. These were then tested using a Deben (Deben, Suffolk, UK) microtensile tester with a 5 N load cell at an extension rate of 1.5 mm/min. The load was applied to the trouser test specimen, and tear force was recorded over a maximum machine extension limit of 11 mm. Figure 1d shows a representative sample in an example of the experimental setup. The tear strength of the full cocoon walls as well as cocoon layers was calculated using eq 1 following ASTM D624,^51^ where *S*_tear_ is the tear strength, *F*_tear_ is the tear force, and *t* is the median thickness of the cocoon wall. All tests were conducted at 65% relative humidity and 21 °C:
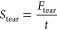
1

The work done in tearing was calculated using eq 2, where *W*_tear_ is the work done in tearing, *F*_tear_ is the tearing force, and Δ*c* is the tearing length:
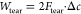
2

The tearing energy was calculated using eq 3. Here, *E*_tear_ is the tearing energy, *F*_tear_ is the tearing force, and *t* is the median thickness of the cocoon wall.^52^
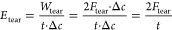
3

Relative density, ρ_r_, and porosity, ϕ, were calculated by using eqs 4 and 5, respectively. Here, ρ is the apparent density of the cocoon wall calculated using the formula  (where *m* is mass and *V* is volume) and ρ_s_ is the density of *B. mori* silk fiber.^53^
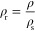
4
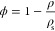
5

### Digital Microscopy and Image Analysis

To develop our microstructural understanding of tear damage, individual cocoon layers were optically examined using a Dino-Lite digital AM4115-FUT microscope (New Taipei City, Taiwan). This microscope possesses a 1.3 megapixel image capture capability, offering a versatile magnification range from 20 to 220×. In this paper, images were captured at magnifications up to 60×. Customised LEDs emitting UV light in the 375 nm spectrum were used to illuminate the samples. To ensure optimal picture quality and precise control over image acquisition, the microscope was securely affixed to a Dino-Lite RK-10 pedestal, as it ensured stable positioning during image capture.

## Results and Discussion

### General Observations

By degumming in accordance with the procedure described in,^32^ the weight percentage of fibroin in native cocoons was determined to be 74% with a standard deviation (SD) of 1.73%. Table 1 presents the physical measurements of 30 cocoons (*n* = 30) in both meridional and equatorial directions. Cocoon walls conditioned under standard temperature (21°) and humidity (55%) were used to determine the apparent density in Table 1.

**Table 1:** *Bombyx mori* Silk Cocoon: Physical Measurements [Including the Range and the Mean ± the Standard Deviation (*n* = 30)]

	cocoon diameter		cut sheet dimensions		sheet thickness (mm)	apparent density of wall (kg/m^3^)
	meridional (mm)	equatorial (mm)	meridional (mm)	equatorial (mm)		
range	29.3–36.9	18.6–23.0	15–22.6	52.0–63.0	0.49–0.92	297–389
mean	32.6 (±2.0)	20.2 (±1.0)	19.4 (±1.7)	56.2 (±2.3)	0.71 (±0.1)	331 (±34)

### Tear Properties of Cocoon Walls

**Figure 2:**
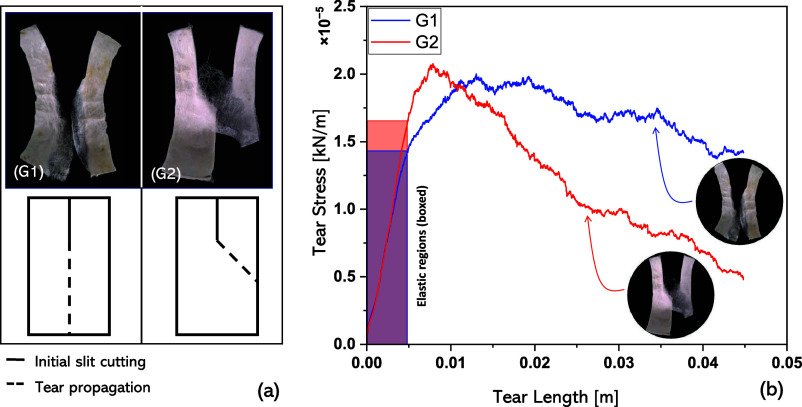
Representative curves from a sample set of 30 cocoons showing tear stress against the tear length for groups 1 (G1) and 2 (G2) generalized tearing orientations. G1 and G2 tearing orientations are shown more clearly on the right-hand side of the figure.

Tear force (*F*_tear_) was determined in accordance with ASTM D624.^51^ This standard computes the highest tear force value from the range of available standards, including: BS2782-3 method 360B,^54^ BS ISO 34-1:2022,^55^ BS EN ISO 6383-1:2015,^56^ ASTM D1938-19,^57^ ASTM D2261^58^ and BS EN ISO 13937-2:2000.^59^ Further details on these standards and comparison curves are provided as an Electronic Supporting Information (Comparison of standard tear force calculations). Using the tear force values (*F*_tear_ = 17.5 ± 4.2 N) from 30 samples (cf. Electronic Supplementary Figure S1), where the thickness (*t* = 0.71 ± 0.12 mm), calculations were made to quantify tear strength (*S*_tear_ = 25 ± 6.1 kN/m), work done in tearing (*W*_tear_ = 9.3 ± 5.5 kN m) and tear energy (*E*_tear_ = 50.1 ± 12.2 kN/m). Two generic tearing characteristics were noted during testing, and these were split into groups 1 (G1) and 2 (G2) and characteristics were equally split in the sample set such that 15 of 30 samples showed G1 characteristics, while the other 15 samples exhibited G2 characteristics. Figure 2a shows pictures of representative torn cocoon walls from G1 and G2 and provides additional schematics to represent the generic tear propagation orientations for each group. G1 cocoon walls tearing was generally oriented in the direction of loading, while the tearing of G2 cocoon walls showed an acute angular orientation to the direction of loading. Of the 15 samples exhibiting G2 characteristics of tear propagation, the angles of orientation were found to range from 25° (minimum) to 58° (maximum), with a mean at 41° ± 14° (SD). Representative tear stress plots are shown for each of the two groups against their tear lengths, Figure 2b. In the G1 plot, a gradual decrease can be observed beyond the maximum tear strength. Dissimilarly, a sharper decline in tear stress can be observed beyond the maximum tearing strength in G2 cocoon walls subjected to trouser tearing.

To assess whether there are any statistical differences between the two groups in terms of their properties, a one-way ANOVA was conducted with significance levels (α) set at 0.001, 0.01, and 0.05. Table 2 provides the results from the one-way ANOVA at α = 0.05. Since the calculated *F*-statistic, *F* = 0.42, is lower than the *F* critical value of 4.2 (the value against which *F* is compared), and the *P*-Value of 0.52 is above α, it can be concluded that there is no difference between the mean values of G1 and G2 at the highest selected α. The results for α = 0.01 and 0.001 were no different, except for that *F* critical increases to 7.6 and 13.5, respectively, further reducing the probability of a type 1 error.

**Table 2:** One-Way ANOVA between Groups 1 and 2 Comparing *S*_tear_, *F* is the *F*-Statistic, SS is the Sum of Squares, MS is the Mean Sum of Squares, *d* is the Degrees of Freedom, and *F*_crit_ is the *F*-Critical Value

source of variation	SS	*d*	MS	*F*	*P*-value	*F*_crit_
between the groups	15.7	1	15.7	0.42	0.52	4.2
within groups	1056.8	28	37.7			
total	1072.5	29				

Additional comparisons between G1 and G2 properties are provided in Table 3, showing the SDs for each sample set, as well as the coefficient of variation (CoV). While it can be noted that the average values of G1 for *t*, *F*_tear_, *S*_tear_, and *E*_tear_ are within one SD from the arithmetic mean of G2 values for *t*, *F*_tear_, *S*_tear_ and *E*_tear_, and vice versa, *W*_tear_ for G1 is within two SDs from the arithmetic mean of *W*_tear_ of G2, and vice versa. Unlike *E*_tear_, the work done (*W*_tear_) is a function of tear length and as such, the comparative distance for tearing is higher in G1 than in G2, a higher value of *W*_tear_ is expected in the G1 samples.

**Table 3:** Comparison of Tearing Parameters among Four Groups with Different Tearing Behaviors

		group 1 (G1)	group 2 (G2)
thickness, *t* (mm)	average	0.72	0.69
	SD	0.13	0.09
	CoV	0.2	0.1
max. force, *F*_tear_ (N)	average	17.1	17.9
	SD	4.2	4.3
	CoV	0.2	0.2
tear strength, *S*_tear_ (kN/m)	average	24.3	25.8
	SD	6.7	5.5
	CoV	0.3	0.2
tear energy, *E*_tear_ (kN/m)	average	48.6	51.5
	SD	13.5	11.0
	CoV	0.3	0.2
work done, *W*_tear_ (N mm)	average	1329	859
	SD	346	438
	CoV	0.3	0.5

**Figure 3:**
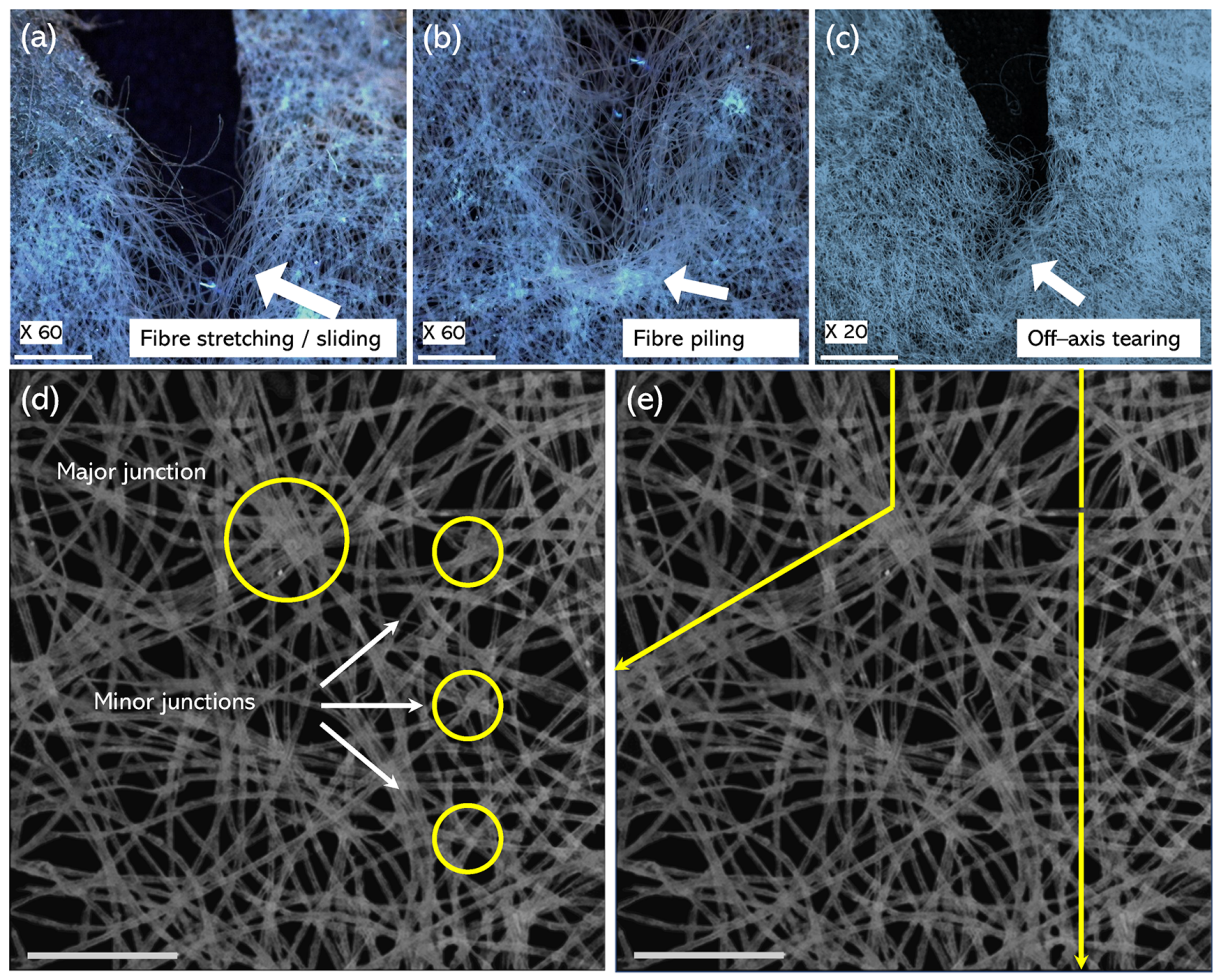
Mechanism of *B. mori* tearing initiates through (a) fiber stretching and sliding followed by (b) comovement of cross fibers adhered to the stretching/sliding fibers resulting in local fiber piling and eventuating in (c) build up of strain energy at fiber piles leading to fracture. Examples of major and minor junctions where fibers cross from layer to layer are shown in (d), while a hypothetical suggestion on how fiber junctions may play a role as nucleation points for differently oriented fiber piling leading to redistributed and reoriented tear energies is shown in (e). All scale bars = 1 mm.

A mechanism for *B. mori* cocoon tearing can be suggested, using images of the specimens torn at different stages, Figure 3a–c. These provide evidence on how tearing both affects, and is affected by, fiber architecture. We find there are three primary fiber failures at the microstructural level that influence the overall mechanism of tearing. In the first Figure 3a, the applied tearing force initiates both fiber stretching and fiber sliding in the direction of the tear. In the second stage Figure 3b, we note that fiber stretching and sliding leads into localized fiber piling, a phenomenon previously reported in mechanically loaded *N. cruentata* spider silk egg cases.^12^ Here, we suggest that similarly to,^12^ cross fibers adhered to stretching/sliding fibers get trapped as they slide into other cross fibers/junctions, creating fiber piles. As fibers pile, the applied tearing force required to cause them to deform, displace, or break is likely to increase as mechanical energy stores within these reduced mobility fibers. These fibers are thus susceptible to fracture rather than displacement under loading as the tearing energy increases. Tearing will therefore propagate at either a different orientation angle to the axis of tear loading Figure 3c, or in the axis of loading, depending on the force vectors (magnitude and direction of force), which in turn are influenced by the local cocoon fiber architectures, Figure 3d,e and the way in which they experience piling. Given that tearing is a function of deformation in the vicinity of the tear tip,^52^ a deviation in deformation from the direction of loading could therefore be associated with the fiber architecture within the cocoon wall. Silkworms build cocoons by overlaying a continuous strand of fiber. This causes fiber crossovers, which are referred to as fiber junctions in this study. As a consequence of this, both larger (major) and smaller (minor) fiber junctions form where fibers are overlaid at different angles. This results in a variable areal density (number of fibers per unit area) as shown in Figure 3d. We suggest that there may be some influence to piling at junctions comprising a higher density of fiber crossovers where there are large numbers of fibers crossing at approximately the same angular orientation, as these may act as nucleation points of some form. Fiber junctions with large numbers of fibers crossing one another would presumably have a greater chance of encouraging the redirection of tearing energies after piling through the nucleation of fibers at these points and resulting in tear line reorientation. Contrarily, where there is a more uniform distribution of fibers with fewer and smaller fiber junctions are perhaps more likely to permit parallelised piling and may encourage tearing in the axis of loading, Figure 3e.

### Tear Properties of Individual Cocoon Wall Layers

**Figure 4:**
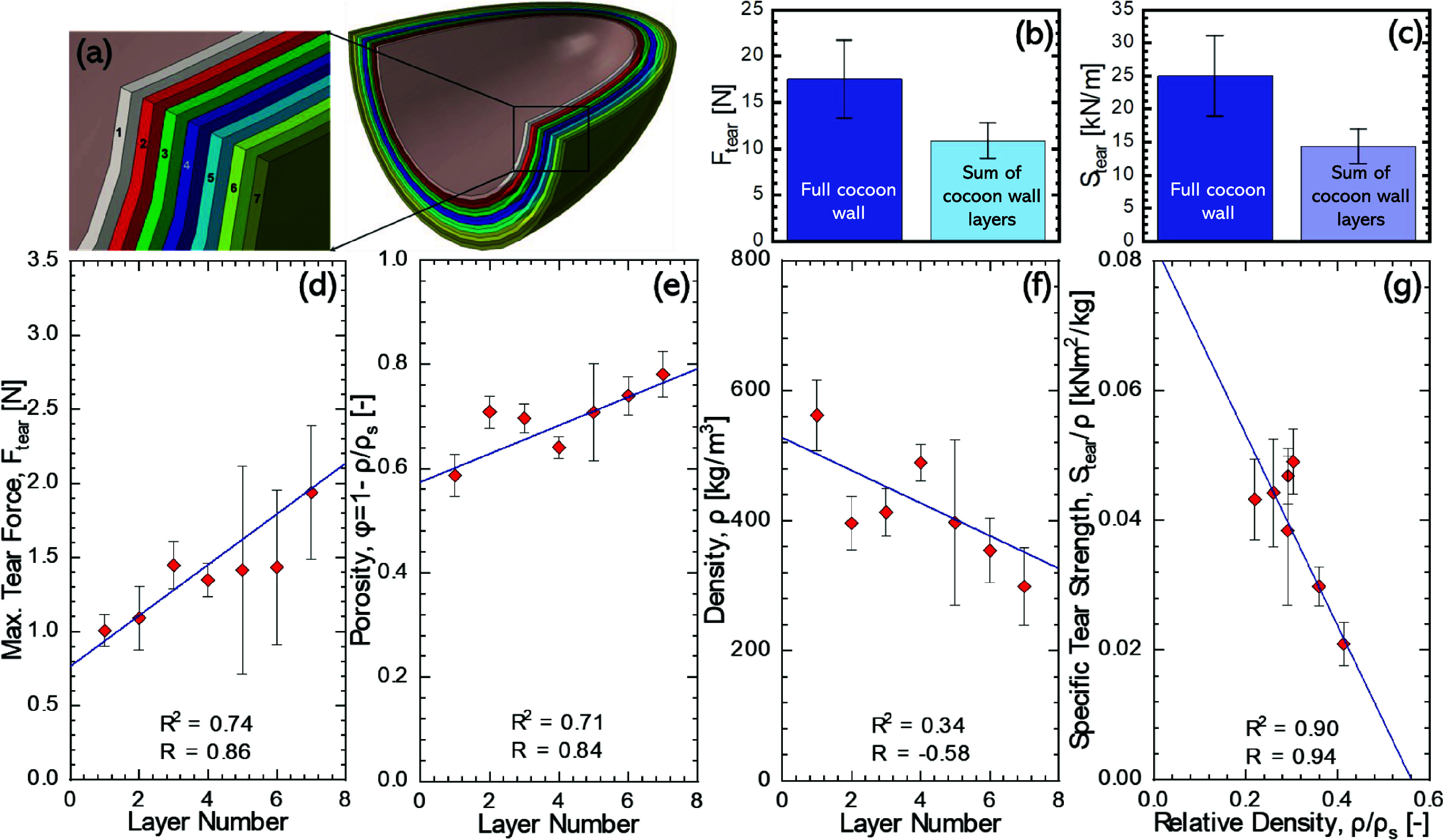
Tear statistics of layers from 1 (inner) to 7 (outer): (a) Layers of *B. mori* cocoon, (b,c) comparison of tearing force and strength for the complete cocoon wall and the sum of the layers (*y*-error bars: standard deviation), (d) tear force, (e) porosity, (f) density, and (g) specific tear strength against the relative density (*y*-error bars: standard error).

Figure 4a illustrates the seven subdivided layers of a *B. mori* cocoon, numbered from one (innermost) to seven (outermost). When comparing the properties of the sum of all cocoon layers against the properties of the cocoon wall, we note that both the tear force (*F*_tear_), Figure 4b, and the tear strength (*S*_tear_), Figure 4c, for the complete cocoon wall, surpass the cumulative values of the individually tested layers. The mean *F*_tear_ of the cocoon wall was measured as 6.6 N higher than the mean sum of *F*_tear_ values for the individual cocoon layers. Similarly, there is an approximately 10 kN/m *S*_tear_ improvement of the complete cocoon wall over the sum of individual layers. The additional resistance to loading in complete cocoon walls is likely attributable to the action of sericin adhering the interfaces of individual layers. The separation of the cocoon into individual layers results in the absence of the additional surface fiber linkages, thus reducing the mechanical properties. Taking the 6.6 N difference therefore as being equally distributed over 6 interfaces in a 7-layer cocoon, we can approximate a 1.1 N overall additional resistance to tearing, per interface. This value exceeds previously reported interlayer peeling loads of 0.32 N^22^ in *B. mori* cocoons, though it should be noted that the earlier reported peeling loads were determined using smaller specimens (20 × 5 mm^2^), distinct experimental speeds, tensile loading, and different mechanical test machines. Furthermore, the present study is specifically focused on tear loading, which localizes any peeling (and thus interfacial effects) to the area near the tear tip.

**Figure 5:**
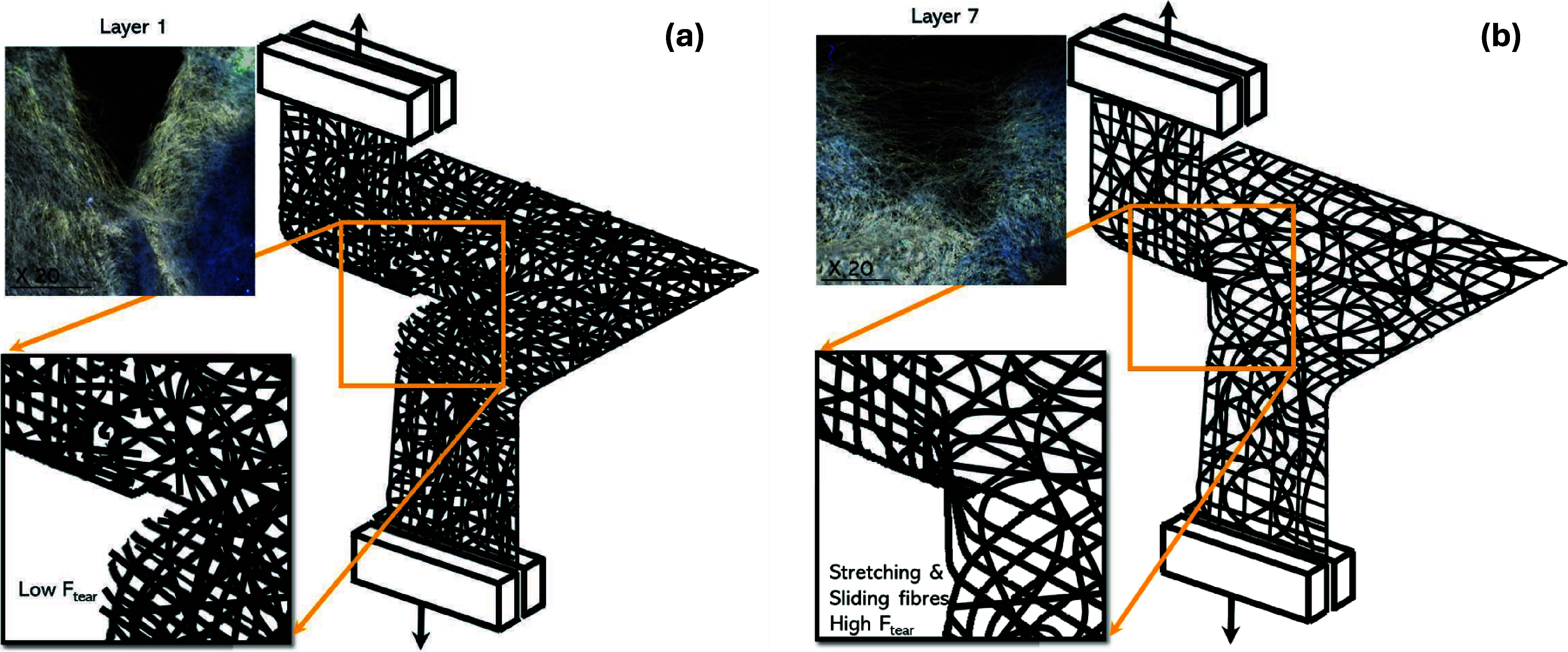
Tearing of individual *Bombyx mori* layers (a) low porosity leading to stress concentrations and low tear force and (b) higher porosity leading to fiber sliding, stretching, and high tear force. Insets: pictures of layer 1 and layer 7, respectively; scale bar = 1 mm.

*F*_tear_ increases linearly from layers 1 to 7 (with Pearson’s correlation coefficient (*R*) at 0.86, and a determination coefficient (*R*^2^) of 0.74), Figure 4d. We note that porosity (ϕ), also increases linearly from layers 1 to 7 starting at 0.59 in the innermost layer to 0.78 in the outermost layer (*R* = 0.84, *R*^2^ = 0.71), Figure 4e. The density of cocoon layers naturally therefore decreases from layers 1 to 7 from 562 kg/m^3^ in the innermost layer to 299 kg/m^3^ in the outermost layer (*R* = −0.58, *R*^2^ = 0.34), as shown in Figure 4f. The progressive increase in the measured tear force from the innermost to outermost layers (0.9 to 2.3 N, respectively), as shown in Figure 4d, can be attributed to increased porosity. This is the opposite trend noted by Chen et al.^22^ for the tensile strength of *B. mori* layers, who note the strength and modulus of the sericin have upper limits of 130 MPa and 3 GPa, respectively. The strength and modulus of silk fiber are comparatively higher, being between 130 and 1410 MPa, and 4.53 and 57.11 GPa, respectively.^22,23^ Chen et al.^22^ note that material failure starts with cracking of the sericin at low strains and the authors attribute this as it has lower breaking strength of sericin as compared with fibers. Notably, the specific tear strength of the cocoon layers exhibited a strong negative correlation with relative density (*R* = 0.90, *R*^2^ = 0.94), as shown in Figure 4g. Given that silk fibers constitute 74% of the cocoon mass, an increase in porosity from the innermost to outermost cocoon layers could be attributed to a reduction in the number of fibers per unit area (the areal density), which is in turn reflected by a decreasing density (cf. Figure 4f). The correlations observed in Figure 4 could be perceived as unusual, since cellular materials subjected to shear will typically show the reverse trends, in that their specific properties will rise, not fall, as a function of increasing relative density.^60^ Nevertheless, there is clear evidence from the literature that torn textiles do behave in this manner.^61−64^ This is because increased porosity in textiles provides additional spaces for fiber stretching, which distributes stress more evenly among neighboring fibers. This resultantly reduces stress concentrations on individual fibers, or small clusters of fibers, thus enhancing the material resistance to tearing. This is schematized in Figure 5. Since each of these layers makes up the full cocoon wall, the mechanism for tearing described earlier (cf. Figure 3) will be comprised of both forms of tearing, allowing the *B. mori* cocoon to have the composite properties of both stretch-dominated failure and brittle fracture. The applied load shifts to the fibers resulting in breakage of fibers at increased strains. The higher density of the innermost layers means there is a greater number of fibers per unit area, which reduces the spaces within fiber architecture, resulting in lower porosity. As a result of the compact fiber packing, fiber movements are restricted and the applied force acts on a single or a smaller number of fibers, Figure 5a. Contrary to this, the increased porosity in the outermost layers provides additional spaces for fiber mobility and stretching, enabling a greater distribution of stress among neighboring fibers and reducing stress concentrations on individual or fewer fibers, thus enhancing resistance to tearing, Figure 5b. In addition, the connectivity model by Chen et al.^65^ informs that cocoon layer properties are governed by porosity. They report that reduced bonding through connectivity will reduce the tensile properties of the cocoon layers. Tearing force is directly proportional to fiber mobility^61−64^ and is in that sense, very different to tension. The morphological structural studies of the cocoon layers conducted by Chen et al.^20,22^ confirmed that the sericin coating does not bond the fibers of the outer cocoon layers as effectively as in the inner layers. In the inner layers, there is better coverage of sericin over the fibroin, resulting in a highly bonded fiber network. The higher porosity coupled to a lower fraction of sericin available for bonding in the outer layers thus allows for greater fiber mobility, and while this aligns with our understanding of how tear strength and porosity are correlated, it also informs that the fraction of sericin and its effectiveness in bonding fibers also contributes to fiber mobility and hence tear resistance. Fibers of the innermost layers are essentially immobile due to both the higher bonding and the higher levels of fiber connectivity, which contributes to the development of stress concentrations at lower strains and consecutive breakage of individual fibers, enabling the propagation of tear.

**Figure 6:**
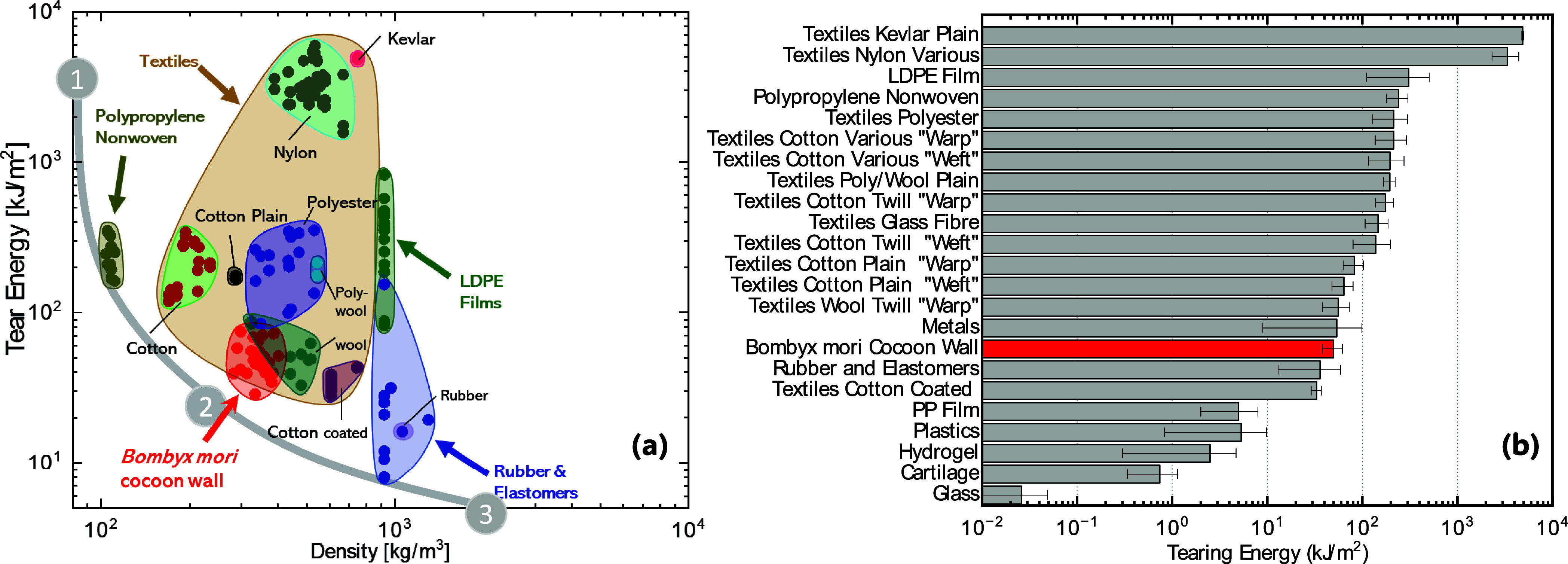
(a) Ashby chart showing the tearing energy of various materials (from^61−64,69−75^) including *Bombyx mori* silk cocoon walls (this study) against density and (b) histogram showing the tear energies (from^61,63,64,69−90^) of a range of different materials/forms including the cocoon walls of *B. mori*. An additional Ashby plot showing tear strength (from^61−64,69−75^) against density including *B. mori* silk cocoon walls (this study) is provided as Electronic Supplementary Figure 2.

It can be useful to know where the tear properties of *B. mori* cocoons fit within the broader spectrum of natural and engineering materials and textiles. Figure 6a shows an Ashby plot comparing *B. mori* tear energies and densities against a variety of materials, including a range of textiles, elastomers, nonwovens, and films. A convex trade-off curve (gray line)^66^ is provided in Figure 6 where (1) identifies an optimal lightweight tear-resistant material and (3) identifies the lower efficiency material that is both heavy and has low tear resistance. Trade-off curves are useful as they elucidate the “good efficiency” areas. In the case of Figure 6 efficiency relates to balanced properties of density and tear resistance, which in this figure is identified as point (2) on the trade-off curve. *B. mori* cocoon walls are therefore neither optimal lightweight tear-resistant materials nor are they inadequate as lightweight tear-resistant materials. Similarly to many other natural materials, *B. mori* cocoon walls therefore exhibit a balanced trade-off between tear resistance and lightweightness.^67,68^ An additional Ashby plot showing tear strength against density is provided as an Electronic Supporting Information Figure 2. Figure 6b compares only the tearing energies of *B. mori* cocoon walls against a range of textiles (in both warp and weft), metals, polymers and films, elastomers, glass, hydrogel, and cartilage. Glass has low values (0.026 kJ/m^2^) due to its inherent brittleness, while metals have tearing energies very similar to those of *B. mori* cocoon walls (50 kJ/m^2^). Textiles due to their interlaced architectures dominate the histogram in terms of their resistance to tear with tougher high-performance materials used in textiles, such as Kevlar and nylons exhibiting the highest tear energies. *B. mori* cocoon walls are natural nonwoven architected fibrous materials and while their tear energies are not as high as systematically organized textiles (such as plain weave, twill weave, etc.), they still show respectable tear resistance, when compared against other natural materials such as cartilage (0.74 kJ/m^2^).

## Conclusions

*B. mori* cocoon walls and their subdivided layers were tested in mode III tearing using trouser test methods. The cocoon wall requires 38% more force to tear than the sum of tearing loads of the seven subdivided layers, and assuming an equal share of interface loading, each interlayer interface contributes 1.1 N of the total tear force. The tear force of seven subdivided layers from the cocoon wall increased consecutively from the inner to the outer layers. Concurrently, the graded architecture of the cocoon wall also increased progressively in porosity and decreased progressively in areal density from the inside layer to the outside layer. These physical properties were found to be associated with increased resistance to tearing forces. Inner layers are less porous and have very little space between the fibers, such that strain energy builds up and the fibers break in succession under an applied force. Fibers in the outer layers have a higher porosity with larger spaces, and this allows for the stretching and sliding of individual fibers, leading to a distribution of strain energy over the neighboring fibers and consequently increasing the resistance of larger pore layers to tear force. A mechanism for the mode III tearing of full cocoon walls is suggested herein, where both fiber sliding and stretching leads to fiber piling, followed by fiber breakage. The orientation of fiber piling determines the angle at which a cocoon wall will tear, and this orientation is presumably related to the presence of fiber junctions that act as nucleation sites for piling. The total failure of the cocoon wall comprising both higher and lower porosity layers is therefore a composite, exhibiting both brittle and stretch-dominated failure mechanisms.

## Data Availability

Data for this publication will be made available through Edinburgh DataShare (https://datashare.ed.ac.uk/) and can also be made available from the corresponding author on request.

## Supporting Information Available

Comparison of standard tear force calculations, tear stress vs. tear length, and tear strength vs. density (PDF)

## Notes

For the purpose of open access, the authors have applied a Creative Commons Attribution (CC BY) license to any author accepted manuscript version arising from this submission.
